# In vitro digestion method for estimation of copper bioaccessibility in Açaí berry

**DOI:** 10.1007/s00706-016-1798-3

**Published:** 2016-07-27

**Authors:** Lena Ruzik, Justyna Wojcieszek

**Affiliations:** Chair of Analytical Chemistry, Faculty of Chemistry, Warsaw University of Technology, Noakowskiego 3, 00-664 Warsaw, Poland

**Keywords:** Amino acids, Bioavailability, Enzymes, Mass spectrometry, Metal complexes

## Abstract

**Abstract:**

Copper is an essential trace element for humans and its deficiency can lead to numerous diseases. A lot of mineral supplements are available to increase intake of copper. Unfortunately, only a part of the total concentration of elements is available for human body. Thus, the aim of the study was to determine bioaccessibility of copper in Açai berry, known as a “superfood” because of its antioxidant qualities. An analytical methodology was based on size exclusion chromatography (SEC) coupled to a mass spectrometer with inductively coupled plasma (ICP MS) and on capillary liquid chromatography coupled to tandem mass spectrometer with electrospray ionization (µ-HPLC-ESI MS/MS). To extract various copper compounds, berries were treated with the following buffers: ammonium acetate, Tris–HCl, and sodium dodecyl sulfate (SDS). The best extraction efficiency of copper was obtained for SDS extract (88 %), while results obtained for Tris–HCl and ammonium acetate were very similar (47 and 48 %, respectively). After SEC–ICP–MS analysis, main signal was obtained for all extracts in the region of molecular mass about 17 kDa. A two-step model simulated gastric (pepsin) and gastrointestinal (pancreatin) digestion was used to obtain the knowledge about copper bioaccessibility. Copper compounds present in Açai berry were found to be highly bioaccessible. The structures of five copper complexes with amino acids such as aspartic acid, tyrosine, phenylalanine, were proposed after µ-HPLC-ESI MS/MS analysis. Obtained results show that copper in enzymatic extracts is bound by amino acids and peptides what leads to better bioavailability of copper for human body.

**Graphical abstract:**

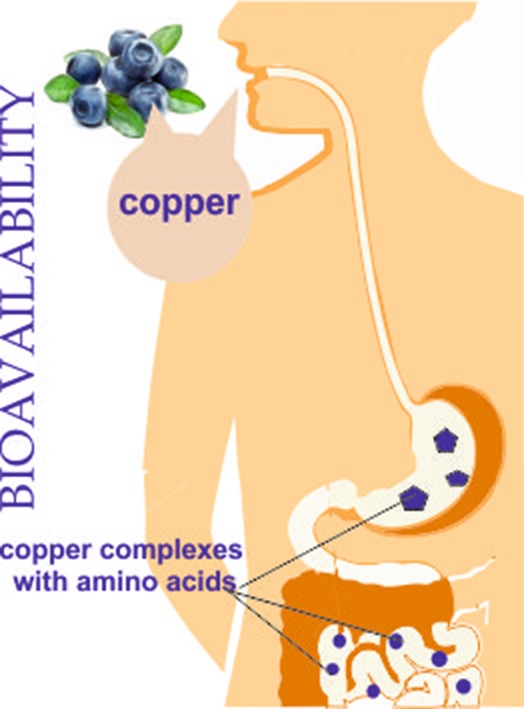

## Introduction

Trace elements play an important role in pathology and physiology of biological system. The existence of living organism is dependent on proper regulation of uptake, assimilation, intracellular compartmentation, and intercellular translocation of trace metals [1]. Trace elements are supplied to the human body primarily from the diet but unfortunately absorption through the diet is not sufficient in most cases [2, 3]. It is relatively easy to determine total concentration of metal using analytical techniques but it does not provide information about its bioavailability [4, 5].

Micronutrients can be absorbed by the human organism in different ways, depending not only on their total concentration but also on their nature and chemical forms [6]. Metals can exist in food in the form of simple ions or in complexes with low, moderate and high molecular mass bioligands, e.g., amino acids, phenolic acids, flavonoids [7]. It was reported in many works that metal chelating peptides derived from a variety of food protein sources (e.g., oyster, hoki, sunflower) have been identified as components with ability to improve the bioavailability of macro and microelements [8–10]. Bioligands can complex metal ions through hydroxyl, carboxylate, and phenolate groups [11, 12]. Metal ions with complexing ligands can create high molecular weight compounds by intramolecular hydrogen binding [13, 14].

The chemical form of metal can influence on whether the metal is beneficial or toxic to human organism [5]. Speciation analysis of trace elements in food is necessary to understand their bioavailability for human organism. Before becoming bioavailable, bioactive compounds must be released from the food and modified during digestion in the gastrointestinal tract [15]. Human digestion is a complex process taking place in the human organism when food is broken into nutrients that can be used by the body [16]. The total concentration of trace elements does not reflect their available amount, only a part of total content is bioavailable for human organism. To determine the bioavailability of metals in food, in vivo and in vitro methods are usually used. In vivo methods provide accurate results but they have a lot of disadvantages, e.g., they are expensive and time consuming. In addition, possibility of measuring some parameters during the experiments is often limited. In opposition to in vivo studies, in vitro experiments are usually simple, low in cost, rapid and additionally they provide an useful alternative to animal and human models using in in vivo studies. The in vitro methods are routinely used to estimate the bioaccessible fractions of trace elements in the diet [4, 17, 18]. The most popular approach of in vitro studies is two-step model that simulate gastric and intestinal digestion using various enzymes. For example, a lot of applicable models are based on incubation with pepsin and pancreatin to simulate gastric and intestinal digestion, respectively [17, 19]. In some cases after in vitro simulation of gastrointestinal digestion, absorption of analyzed elements is simulated by ultrafiltration through filters with molecular mass cut-offs of 3, 10, 30 kDa [20].

Two terms are usually used to describe soluble products present in the gastrointestinal tract. The term ‘bioavailability’ has several working definitions, depending on the research area it applies to. From the nutritional point of view, bioavailability refers to the fraction of the nutrient or bioactive compound ingested that is available for use in physiologic functions or to be stored. On the other words, bioavailability is the amount of low molecular weight compounds obtained during digestion which can access blood stream. Bioavailability is a key concept of nutritional effectiveness, irrespective of the type of food being considered. Bioaccessibility is defined as the fraction of substances which are soluble in the gastrointestinal tract and available for absorption by human body [5, 18, 21, 22]. Knowledge about concentration of element of interest in the bioaccessible fraction is necessary for estimation of bioavailability thus the information on the bioaccessibility of important nutrients in food and food supplements seems to be important. In present work, as a part of ongoing studies of elements bioavailability from human diet, we have tried to estimate the bioaccessibility of copper in the Açaí berry (*Euterpe oleracea* M.) by in vitro simulation of gastrointestinal digestion using pepsin (gastric digestion) and pancreatin (intestinal digestion). The second aim of the investigation was the identification of bioligands complexing copper, which are responsible for higher bioavailability of analyzed trace element for human body. This is, to our knowledge, first attempt to estimate bioaccessibility of trace element necessary for human being in Açaí berry, known as a “superfood”.

## Results and discussion

### Total concentration of copper in samples of Açaí berry

Total concentration of copper in Açaí berry was determined by means of ICP MS. Results represent average amount established for three samples (each measured three times) and show that total concentration of copper was 23.9 ± 2.6 µg g^−1^ (RSD 1.3 %). These results are in good agreement with literature data reported for lyophilized fruits [23].

The amounts of copper were measured by ICP MS also for solutions obtained by buffer extraction and simulation of gastrointestinal digestion of Açaí samples. The efficiency of extraction of copper after buffer and enzymatic extraction is presented in Table 2. The  % of bioaccessibility of the copper in Açaí was calculated on the basis of copper concentration in gastric and gastrointestinal digests, by means of following formula:

$${\text{\% Bioaccessibility}} = \frac{{\left[ {\text{GC}} \right] {\text{or [GIC}}]}}{{ [ {\text{TC}}]}}\; \times \;100\;\%$$

where [GC] = content of copper in gastric digest, [GIC] = content of copper in gastrointestinal digest, and [TC] = total concentration of copper in Açaí sample.

The results show that after gastric digestion copper is extracted in more than 80 % of total content what could indicate that proteins are the main ligands binding copper in Açaí. After the end of simulation of digestion, bioaccessibility increased to about 100 % what leads to the conclusion that all copper compounds present in Açaí are bioaccessible for human body. Obtained results can lead to the conclusion, that Açaí berries can be treated as a natural source of copper. As a consequence, Açaí berries are a great alternative to synthetic diet supplements created on the chemical way. It is especially important due to the fact that synthetic supplements of minerals with similar chemical characteristics could reduce copper absorption. Bioligands such as proteins, present in a significant amount in Açaí berries, tend to improve copper absorption and bioavailability by enhancing its solubility [24]. Comparing to other studies of copper bioaccessibility found in the literature, different results were obtained depending on analyzed kind of food. Bioaccessibility from commercial bee honey was about 96 % [25]. This high result might be due the fact that copper in analyzed honey probably does not exist in the form of complicated structures and compounds with lower molecular mass are better accessible for human body. After analysis of molluscs, determined copper bioaccessibility was in the range of 54–96 %. Different results were obtained because of various kind of analyzed molluscs and their form (raw or steam) [26]. Results obtained after analysis of grain tea-biscuits samples led to the conclusion, that copper bioaccessibility was about 50–70 % [27]. The lowest bioaccessibility of copper was calculated after analysis of Turkish hazelnuts (17–31 %). It was explained by the presence of nutrients with the same chemical characteristics, what led to reduction of copper absorption [22]. In the case of buffer extracts, the best result was obtained for sodium dodecyl sulfate. The presence of SDS significantly improves the efficiency of extraction (88.2 %) what could indicate that copper creates complexes mainly with hydrophobic proteins extracted by means of SDS solution. For Tris–HCl and ammonium acetate extracts, the efficiency of copper extraction was very similar (47.1 and 48.0 %, respectively) (Table 1).

**Table 1:** Efficiency of copper extraction from buffer and enzymatic extracts of Açaí berry

	Extraction efficiency/%	Bioaccessibility/%
10 mM ammonium acetate pH 7.4	48.0 ± 0.5	
30 mM Tris–HCl pH 7.4	47.1 ± 0.3	
2 % (w/v) SDS in water	88.2 ± 0.8	
Gastric digestion (pepsin)		81.2 ± 1.3
Gastrointestinal digestion (pancreatin)		103.7 ± 2.6
Total content of copper/µg g^−1^	23.9 ± 2.6	

The RDA values for minerals provided by the European Commission (European Communities, 2008) were used to check if the daily dosage of Açaí would provide a significant contribution to the diet. The daily dosage of Açaí contributes to the RDA approximately 25 % for copper (recommended daily dosage of copper is 1.0 mg for adults). According to the results reported in this work, berries are a good source of copper and could contribute to meeting the copper requirements for human body.

### SEC-ICP MS characteristics of copper compounds extracted from berries samples by different extraction media

**Fig. 1:**
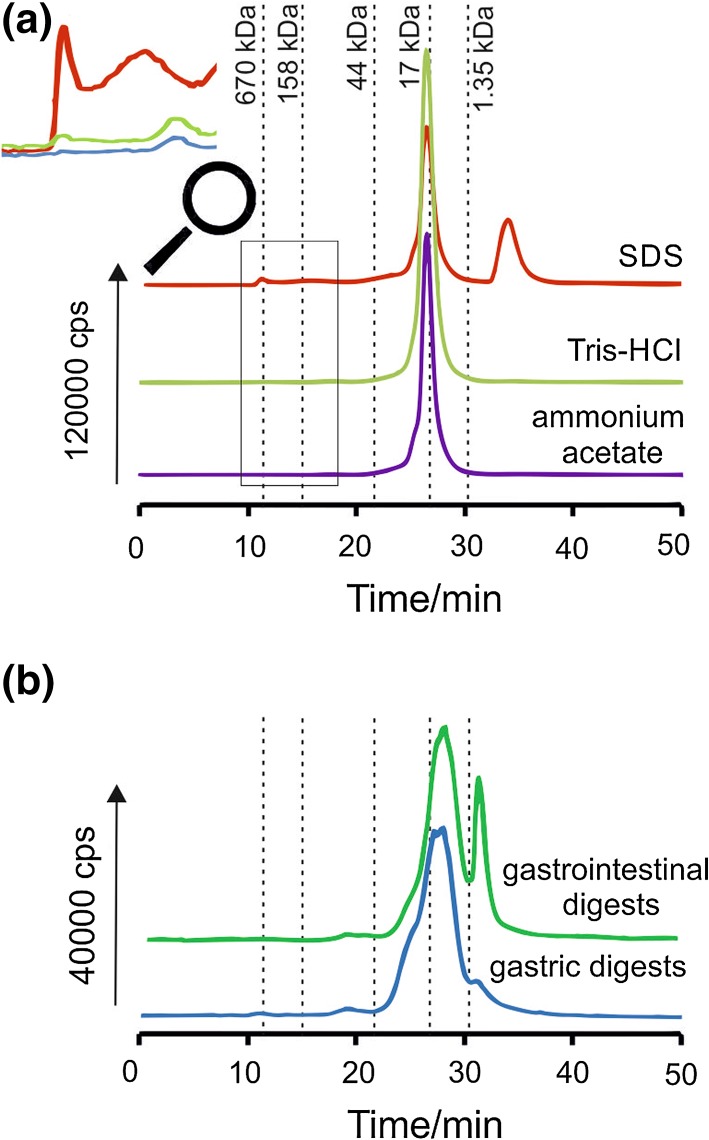
SEC-ICP MS chromatograms obtained after analysis of: **a** buffer extracts (SDS, Tris–HCl, ammonium acetate), **b** enzymatic extracts (pepsin—gastric digestion, pancreatin—gastrointestinal digestion)

Size exclusion chromatography in coupling with inductively coupled plasma mass spectrometry allows separating and element-specific detection of eluted species. The SEC-ICP MS chromatograms obtained after analysis of Tris–HCl, SDS, and ammonium acetate extracts of Açaí consist of one main peak at *t*_*r*_ = 26.5 min (Fig. 1a). On the basis of the column calibration, this peak corresponds to copper complexes with bioligands of molecular mass about 17 kDa. On the chromatogram obtained for SDS extract three additional peaks could be observed, corresponding probably to compounds of copper with hydrophobic proteins. The first peak at about 11 min was eluted in void column volume, corresponding to high molecular weight compounds with molecular mass >650 kDa, what could be explained by the ability of metal ions to create agglomerates even with low molecular weight ligands. The small peak was observed after 16 min of analysis, which can correspond to fraction of copper compounds with molecular mass about 150 kDa. The next main peak, characteristic for SDS extract, was obtained at 34 min and it could correspond to complexes at molecular mass lower than 1.4 kDa. Except peak at 26.5 min, one small, additional peak was observed at 18 min in the case of Tris–HCl and ammonium acetate extracts. It corresponds to complexes of copper with compounds at molecular mass between 160 and 44 kDa. The extraction by ammonium acetate and Tris–HCl should lead to the release of metal complexes with organic acids and hydrophilic proteins or peptides, respectively. Chromatographic profiles obtained for those extracts are almost identical, what could suggest that Tris–HCl and ammonium acetate are able to extract compounds of similar molecular weight and hydrophilic character. Detailed information about extracted compounds could be obtained by using more advanced separation techniques. The results obtained using size exclusion chromatography suggest the presence of different complexes of copper with peptides and proteins, extracted by chosen solutions.

### Estimation of bioaccessibility of copper compounds by SEC-ICP MS profiling of gastric and gastrointestinal extracts

The SEC-ICP MS analysis of extracts obtained after digestion with both pepsin and pancreatin were carried out with the same conditions as for analysis of Tris–HCl, SDS, and ammonium acetate extracts. Chromatograms obtained after analysis of enzymatic extracts of Açaí consist of two peaks with various intensity (Fig. 1b). The first peak at 27 min, present on both chromatograms, corresponds to compounds at molecular mass between 17 and 1.35 kDa, probably from copper adducts with products of digestion of proteins present in analyzed berry. This signal can be observed also due to copper binding by enzymatic proteins used during digestion or their self-digestion residues. The ability of proteins and peptides to interact with metals and improving their bioaccessibility was already reported in earlier studies [8]. The second peak, obtained at 31 min, was significantly higher in the case of gastrointestinal extract in comparison with gastric extract obtained by the action of pepsin. On the basis of the column calibration, this region represents copper compounds with molecular weight less than 1.35 kDa. This second peak of gastrointestinal extract may correspond to copper complexes with products of digestion of polysaccharides, which were created because of action of amylase present in pancreatin cocktail. The high content of polysaccharides in Açaí was already confirmed in a previous report [28]. Results obtained after analysis of enzymatic extracts suggest the presence of copper complexes with low molecular weight compounds, probably with peptides and amino acids or monosaccharides created in the gastrointestinal tract during digestion process. However, the effectiveness of the supplementation cannot be certain until the nature of the bioligands complexing metals (peptides, polysaccharides, organic acids) will not be clarified. Therefore, it is of special interest to get the knowledge, what kind of peptides or amino acids present in Açaí or created during digestion, are involved in copper binding.

### Identification of copper complexes by µ-HPLC-ESI MS/MS analysis

**Fig. 2:**
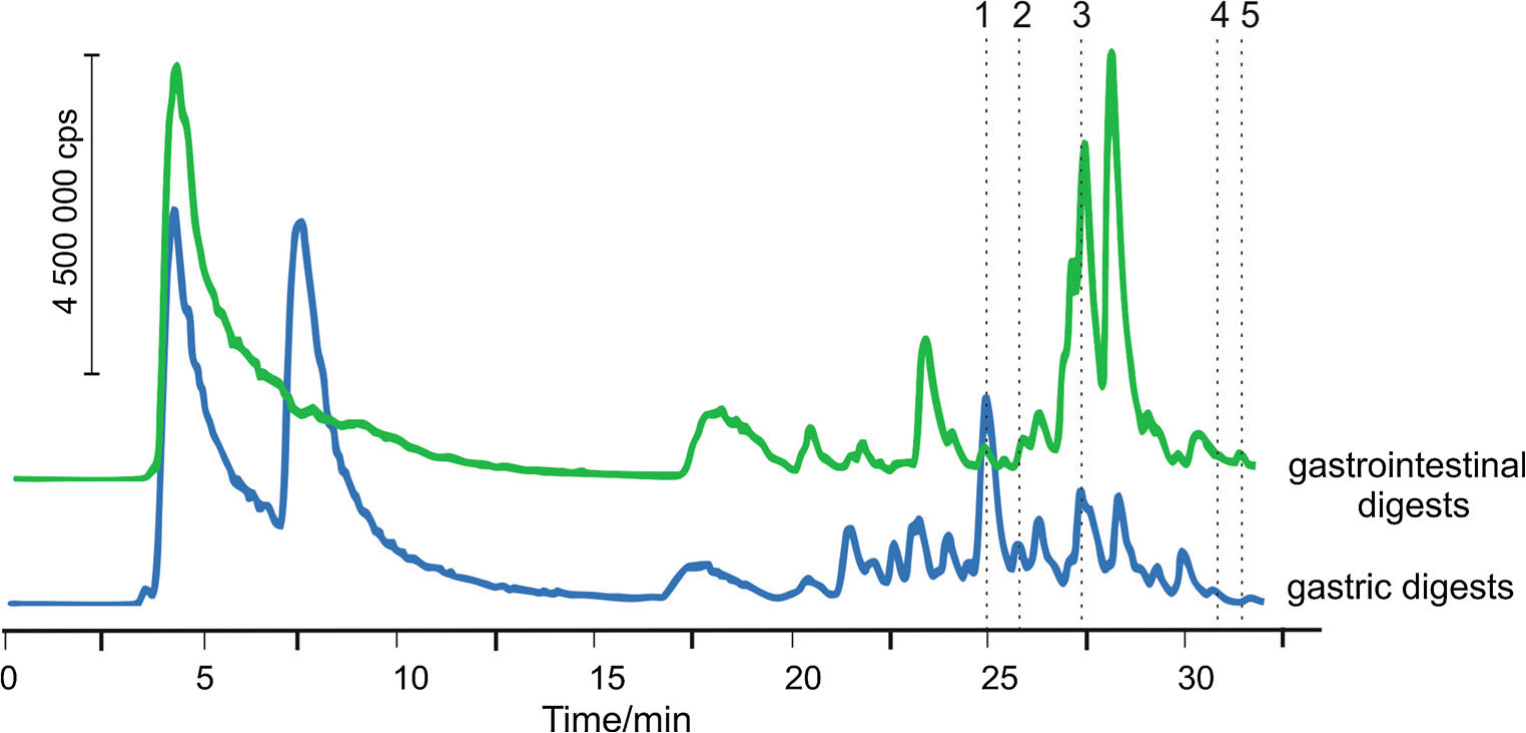
µ-HPLC-ESI MS chromatograms registered in negative ion mode, obtained after analysis of enzymatic extracts (after simulation of gastrointestinal digestion) of Açaí berry

The extracts obtained after simulation of gastrointestinal digestion were ultrafiltrated using 3 kDa cut-off filters and analyzed by µ-HPLC-ESI MS/MS technique. Elution was performed with the gradient program using 0.15 % (v/v) formic acid in water and methanol as eluents. The presence of a formic acid used as an acidic modifier essentially affected the sensitivity of ESI MS detection. All mass spectrometric data were recorded in positive and negative ion modes in the range of *m/z* from 50 to 1000. The mass spectra obtained for all chromatographic peaks registered in both ionization modes were searched for isotopic pattern corresponding to copper ion. After studies of the mass spectra, signals with isotopic profile characteristic for single charge complexes with one copper were found. The procedure for identification of copper complexes with peptides and amino acids was based on the fragmentation of signals pre-characterized by distinctive comparison of experimental and theoretical isotopic profiles of the supposed copper compounds. On the obtained chromatograms five peaks with isotopic pattern specific for copper were observed in enzymatic extracts. Signals 1–3, obtained from copper compounds (Fig. 2) could be registered when ESI MS detection was performed in positive and negative ion mode and compounds 4 and 5—exclusively in negative ion mode.

The MS signals with isotopic profile for copper (^63^Cu—69 %, ^65^Cu—31 %) were attributed to the quasi-molecular ions, then they were fragmented by collision-induced dissociation and analyzed in product ion mode. Structures of identified copper complexes and fragmentation paths proposed after analysis of obtained fragmentation ions are presented in the Table 2. All identified compounds of copper were eluted after 24 min of chromatographic process what could indicate that they are mostly hydrophobic. This is in good agreement with results obtained after SEC-ICP MS analysis, when the majority of copper complexes were extracted using SDS solution, dedicated for extraction of hydrophobic compounds. Longer retention times indicate the presence of amino acids with aromatic or heterocyclic rings in the structure which are responsible for hydrophobic interactions with stationary phase. The first mass spectrum was observed at 24.8 min only in gastric extract, it was not appeared in extract obtained after gastrointestinal digestion. After analysis of fragmentation ions the compound was identified as copper complex with phenylalanine and aspartic acid. The value *m/z* = 247 can indicate copper binding by phenylalanine after the loss of aspartic acid, while characteristic value at *m/z* = 213 can correspond to the residue of phenylalanine bound to copper. The signal at *m/z* = 86 does not consist isotopic profile specific for copper and it could correspond to the residue of aspartic acid. The next mass spectra were obtained for parent ions at *m/z* = 302 and 300, at 25.6 min. This signal was observed after analysis of both gastric and gastrointestinal extracts. After analysis of fragmentation ions, complex of copper with glycine and phenylalanine was proposed as the compound of copper accessible for human organism. The signal at *m/z* = 227 could correspond to copper compound with phenylalanine and signal at *m/z* = 199 could be obtained after the loss of carbonyl group. For the signals at *m/z* = 86 and 72, the isotopic profile of copper was not observed. The next fragmentation was carried out for signal at *m/z* = 330 ([M − H]^−^) which was observed at 27.1 min, for both enzymatic extracts. Fragmentation was carried out in the negative mode and, in consequence, signals at *m/z* = 185 and 155 appeared in the mass spectrum, both with the presence of isotopic pattern characteristic for copper. Analysis of all the signals led to propose of the complex of copper with peptide His-Asp as the most probable candidate. Next fragmentation was carried out for the ion at *m/z* = 483 which was appeared at 31.5 min, observed only in gastric digest and exclusively in negative ion mode. The molecular mass can indicate the presence of a tyrosine containing a phenyl group which can be easily deprotonated. The signal was identified as corresponding to copper complex with 3 different amino acids—tyrosine, threonine, and serine. Next signal, obtained at 31.9 min of chromatographic separation, was registered in the negative ion mode and was present in both gastric and gastrointestinal extracts. The most characteristic value *m/z* = 385 corresponds to the loss of CO_2_ from carboxyl group. Next fragment obtained at *m/z* = 298 could indicate the loss of alanine, while another fragmentation ion at *m/z* = 225 can correspond to copper complex with phenylalanine. Phenyl group can be additionally responsible for the longer retention time. The isotopic pattern characteristic for copper was not found at *m/z* = 130. Described compound was identified as copper complex with alanine, phenylalanine, and valine.

**Table 2:** Copper complexes observed in µ-HPLC–ESI–MS/MS spectra after analysis of enzymatic extracts of Açaí berry

No.	*t*_R/_min	*m/z* [M + H]^+^/[M − H]^−^	Proposed structure	Gastric/gastrointestinal digestion	Product ions (MS/MS)
1	24.8	360^a^/358^a^	Phe-Cu-Asp	+/−	(ESI+) 247^a^ (Phe-Cu + H_2_O), 213^a^ (Phe-Cu − H_2_O + H_2_), 86 (C_4_H_7_NO)
2	25.6	302^a^/300^a^	Gly-Cu-Phe	+/+	(ESI+) 227^a^ (Phe=Cu), 199^a^ (Phe-Cu − CO), 86 (C_4_H_7_NO), 72 (C_4_H_9_N)
3	27.1	332^a^/330^a^	His-Asp=Cu	+/+	(ESI−) 185^a^ (His = Cu − 2H_2_O + 3H_2_), 155^a^ (C_2_H_3_NO = Cu + 2H_2_O)
4	31.5	ND/483^a^	Tyr-Cu-Ser-Thr + 2H_2_O	+/−	(ESI−) 447^a^ (483^a^ − 2H_2_O), 390^a^ (447^a^ − C_2_H_3_NO), 273^a^ (390^a^ − Thr + H_2_)
5	31.9	ND/429^a^	Val-Cu-Phe-Ala	+/+	(ESI−) 385^a^ (429^a^ − CO_2_), 298^a^ (385^a^ − Ala + H_2_), 225^a^ (Phe=Cu), 130 (C_6_H_9_NO_3_)

^a^Signals with isotopic pattern characteristic for copper ion

The presented study demonstrated that peptides and amino acids formed during degradation of proteins by the action of pepsin and pancreatin are able to chelate copper ions and, in consequence, improve copper bioavailability. It was already reported that compounds of copper binding by peptides lead to better bioavailability of analyzed element by human organism [29].

## Conclusions

Due to the lack of information about bioavailability of metals in different kind of food belongs to the category “superfoods”, the main goal of presented study was to estimate bioaccessibility of copper complexes in Açaí berry using two-step model of in vitro simulation of gastrointestinal digestion. It was first attempt to estimate bioaccessibility of copper complexes in Açaí berry using mass spectrometry techniques.

Elution profiles of copper complexes extracted by different buffers and analyzed by SEC-ICP MS were quite similar and consisted of one main peak corresponding to copper compounds at molecular mass about 17 kDa. The efficiency of extraction was calculated by the ratio of copper amount in buffer extract to its total concentration. Results show that the most amount of copper was extracted by means of SDS solution (88 %). Four signals observed after analysis of SDS extract coming probably from complexes with hydrophobic proteins suspected to reveal ability to bind copper.

Results of the in vitro gastrointestinal digestion have indicated that contribution of the soluble fraction of copper to total concentration of analyzed element is about 100 % what could mean that copper compounds present in Açaí are potentially available for human body. It can lead to the conclusion, that Açaí berries can be treated as a natural source of copper supplementation, instead of synthetic diet supplements. Signals obtained during SEC-ICP MS analysis demonstrated that copper complexes extracted by the simulation of gastrointestinal corresponding probably to copper adducts with products of digestion of proteins. Moreover, signal obtained only after analysis of gastrointestinal extract may correspond to copper complexes with products of digestion of polysaccharides, which could be created because of action of amylase present in pancreatin cocktail.

Extracts obtained during simulation of digestion were analyzed using µ-HPLC-ESI MS/MS method. MS/MS spectra, obtained as the result of fragmentation of selected quasi-molecular ions allowed identification of fragments lost during collision-induced degradation of copper complexes and, in consequence, it was possible to propose their structures. Amino acids such as phenylalanine, tyrosine, aspartic acid, valine, threonine, and serine were shown to be able to form complexes of different hydrophobicity and solubility. Results obtained after µ-HPLC-ESI MS/MS analysis show that copper in enzymatic extracts is bound by amino acids and peptides what leads to better bioavailability of copper in human body. The methods described in this work may be used as simple way to study metal and metal complexes in different kind of food products.

## Experimental

Pepsin from porcine gastric mucosa and pancreatin were of biological grade (Sigma-Aldrich, Buchs, Switzerland). Sodium chloride, ammonium acetate, sodium dodecyl sulfate, tris(hydroxymethyl)aminomethane, hydrogen peroxide, sodium hydrogen carbonate, and hydrochloric acid were also purchased from Sigma-Aldrich and were of analytical reagent grade. Nitric acid was the product of Fluka (Switzerland) of purity for trace metals analysis. Formic acid of LC/MS purity was obtained from Fisher Scientific (Fair Lawn, NJ, USA). Methanol (LC–MS grade) was purchased from POCH (Gliwice, Poland). Deionized water (18 MΩ cm) prepared with a Milli-Q system (Millipore Elix 3, Millipore, Saint-Quentin, France) was used throughout. The SEC column was calibrated using size exclusion standard (BIO-RAD, Warsaw, Poland).

### SEC-ICP MS

Prepared samples were analyzed on a Superdex200 10/300GL (GE Healthcare Life Sciences) exclusion column. Chromatographic separations were performed using Agilent 1100 gradient HPLC pump (Agilent Technologies, Waldbronn, Germany) as the sample delivery system. Elution was carried out isocratically at 0.7 cm^3^ min^−1^ using 10 mM ammonium acetate buffer (pH 7.4) as the mobile phase. Solvent flow from chromatographic system was introduced into the ICP MS by means of PEEK tubing. Before the analysis the column was calibrated with a mixture of thyroglobulin (670 kDa), γ-globulin (158 kDa), ovalbumin (44 kDa), myoglobin (17 kDa), vitamin B_12_ (1.35 kDa).

An Agilent 7500a ICP Mass Spectrometer (Tokyo, Japan) was used as an on-line detector in SEC-ICP MS analysis and as an element-specific detector for quantification of metal content in berries samples. A Ni/Cu skimmer was installed in the interface, the position of torch and nebulizer gas flow was adjusted daily with special emphasis to decrease the level of CsO^+^ below 0.2 % with the aim to minimize the risk of polyatomic interferences caused by oxides. The working conditions were optimized daily using a 10 µg dm^−3^ solution of ^7^Li^+^, ^89^Y^+^, and ^209^Bi^+^ in 2 % (v/v) HNO_3_.

### µ-HPLC-ESI MS/MS

Capillary HPLC-ESI MS/MS analyses were performed by means of Agilent 1200 series chromatograph (Agilent Technology, Waldbronn, Germany) equipped with a binary capillary pump, degasser, autosampler and thermostatically controlled column compartment. Separation of the metal complexes was carried out using capillary Zorbax SB C18 column (4.6 mm × 150 mm, 5.0 µm). Column was coupled to triple quadrupole mass spectrometry.

Identification of compounds of interest was carried out by means of electrospray ionization triple quadrupole mass spectrometer (Agilent 6460 Triple Quad LC/MS, Agilent Technologies, Santa Clara, CA, USA), working in scan or product ion mode. All the operations, acquiring and analysis of data were processed by MassHunter Software (Agilent Technology, USA). Operational parameters are summarized in Table 3.

**Table 3:** Operational parameters for HPLC, ICP-MS, and ESI–MS

Settings		
SEC separation		
Pump	Agilent 1100	
Column	Superdex 200 (10 × 300 mm × 10 µm) − GE Healthcare Life Sciences	
Mobile phase	10 mM ammonium acetate buffer (pH 7.4)	
Elution program	isocratic	
Flow	0.7 cm^3^ min^−1^	
Injection volume	100 mm^3^	
Column temperature	24 °C	
µ-HPLC separation		
Pump	Agilent 1200 Series	
Column	Zorbax SB C18 (5.0 µm, 4.6 × 150 mm)	
Injection volume	0.1 mm^3^	
Flow rate	5 mm^3^ min^−1^	
Eluents	A: 0.15 % (v/v) formic acid in water B: methanol	
Gradient program	Time/min	%B
	0	5
	8	5
	23	100
	35	100
ICP-MS	Agilent 7500a	
RF power	1350 W	
Plasma, auxiliary, nebulizer gas flow	15.0, 1.0, and 1.05 dm^3^ min^−1^	
Cones	Sampler—Pt, Skimmer—Ni	
Monitored isotopes	^63^Cu, ^65^Cu	
Dwell time	0.1 ms	
ESI–MS/MS detection	Agilent 6460 Triple Quad LC/MS with JetStream technology	
Polarity	Positive, negative	
Mode	SCAN, PI	
Ionization voltage/V	2500(PI), 1500(NI)	
Nebulizer pressure/psi	55	
Gas temperature/°C	300	
Gas flow/dm^3^ min^−1^	8	
Sheath gas flow/dm^3^ min^−1^	6	
Sheath gas temperature/°C	300	
Mass range/*m/z*	50–1000	
Collision energy/eV	5–30	

### Other apparatus

A Bandelin Sonorex Model 1210 ultrasonic bath (Germany) and MPW Model 350R centrifuge (MPW Warsaw, Poland) were used for extraction procedures. Microwave digestion Speedwave^®^four Berghof, (Germany) was used for mineralization of Açaí berry. A thermostatically controlled water bath (Memmert WB 10, Germany) was used during simulation of digestion to samples incubation.

### Sample mineralization for determination of total amount of metals

The lyophilised Brazilian Açaí berry was obtained from Kenay (Poland—imported from Brazil) and stored at room temperature until sample preparation.

For determination of total content of elements three samples (0.3 g of dry mass) were digested using microwave-assisted mineralization with a mixture of 5 cm^3^ of 69 % HNO_3_ and 3 cm^3^ of 30 % H_2_O_2_. After cooling down, obtained solutions were diluted to a final volume of 25 cm^3^ with MQ water. Further dilutions were prepared directly before ICP MS analysis with 2 % (v/v) HNO_3_ and 10 ng cm^−3^ of yttrium as an internal standard. Multi-element standard solution was chosen for calibration. The calibration graphs were obtained for metal concentration in the range of 0.1–60.0 ng cm^−3^. Calibration curves were linear in the investigated range from 2.0 to 60.0 ng cm^−3^ with *r*^2^ above 0.998. Limits of detection (LOD) were calculated for standard deviations (SD) of 10 measurements for blank and it was found to be 0.3–1.0 ng cm^−3^.

### Fractionation studies of element species

Homogenized samples of Açaí berry were extracted with the following buffers: 10 mmol dm^−3^ ammonium acetate (pH 5.5) for extraction of organic acids and other small bioligands suspected to reveal ability to bind copper, 30 mmol dm^−3^ Tris–HCl (pH 7.4) dedicated to water soluble metal complexes and 2 % sodium dodecyl sulfate (SDS) in water dedicated to hydrophobic proteins, which were suspected to create complexes with the investigated element [30].

Samples of Açaí berry (0.05 g of dry powder) were extracted with 1.0 cm^3^ of each solvent for 1 h using ultrasonic bath. After sonication, the obtained solutions were centrifuged for 20 min at 15,000 rpm at 15 °C. The final supernatants were filtered with 0.45 µm syringe filter (Sigma-Aldrich, Bellefonte, PA, USA), two first drops were discarded and only the remaining part of the filtrates were injected on the size exclusion column.

### In vitro gastrointestinal digestion studies

The in vitro digestion method was based on that described by Luten et al. [31], modified and adapted for the berries samples being studied. The simulation of gastric digestion was initiated by adding 2.5 cm^3^ of freshly prepared pepsin solution (6 % w/v pepsin in 0.15 mol dm^−3^ NaCl, acidified by HCl to pH 1.8) to 0.07 g of lyophilized berries. The mixtures were sonicated for 15 min for initial degassing and then incubated in a thermostatic water bath for 3 h at 37 °C, with continuously stirring at a low speed. In the next step, the mixtures were centrifuged at 4 °C for 20 min at 10,000 rpm. The supernatants were filtered through 0.45 µm syringe filters (Sigma-Aldrich, Bellefonte, PA, USA).

**Fig. 3:**
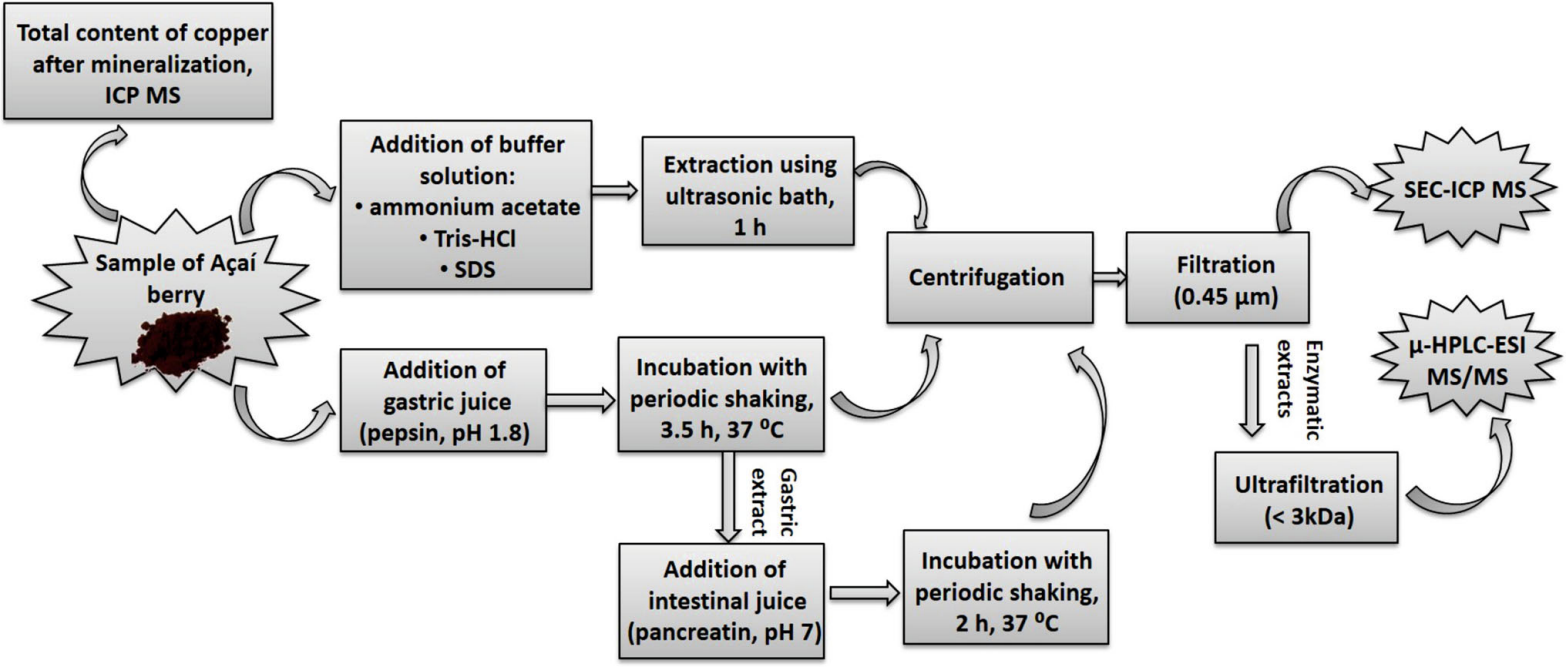
The scheme of buffer and enzymatic extraction of copper complexes from Açaí berry

For intestinal digestion, the pH of the extracts obtained after gastric digestion was adjusted to neutral pH by addition of NaHCO_3_ solution. Afterwards, 2.5 cm^3^ of freshly prepared pancreatin solution (1.5 % w/v pancreatin in 0.15 mol dm^−3^ NaCl) was added to the gastric digests and sonicated for 15 min in an ultrasonic bath. The mixtures were incubated in thermostatic water bath for 2 h at 37 °C, with continuously shaking. After gastrointestinal digestion, the samples were sonicated for 20 min and centrifuged at 10,000 rpm for 20 min. The supernatants were filtered through 0.45 µm syringe filters. In the next step, gastric and gastrointestinal extracts were ultracentrifuged through filters with molecular mass cut-off of 3 kDa. It allows to separate enzymatic proteins from small molecular weight compounds [32]. Additionally, the efficiency of copper extraction was estimated by establishing the amounts of elements in extracts after buffer and enzymatic extraction against total concentration of elements in mineralized samples. The procedure of samples preparation is presented in Fig. 3.
